# Deciphering 6-mer Spectra Distribution Rules in Coronavirus Genomes: Application to Comparative Genomic Analysis

**DOI:** 10.3390/ijms27083604

**Published:** 2026-04-18

**Authors:** Zhenhua Yang, Hong Li, Xiaolong Li, Guojun Liu

**Affiliations:** 1 School of Economics and Management, Inner Mongolia University of Science & Technology, Baotou 014010, China; 2 Inner Mongolia Industrial Informatization and Industrial Innovation Research Center, Inner Mongolia University of Science & Technology, Baotou 014010, China; 3 Laboratory of Theoretical Biophysics, School of Physical Science & Technology, Inner Mongolia University, Hohhot 010021, China; 4 College of Science, Inner Mongolia Agriculture University, Hohhot 010018, China; 5 School of Life Science and Technology, Inner Mongolia University of Science & Technology, Baotou 014010, China

**Keywords:** coronaviruses, distribution rules of 6-mer spectra, CG-specific selection mode, phylogenetic relationships, the whole-genome level

## Abstract

Given the rapid mutation and high transmissibility of coronaviruses, especially SARS-CoV-2, comparative genomic studies are crucial for understanding viral evolution, transmission dynamics, and therapeutic development. In prior work, we analyzed and compared the spectral distribution patterns of various k-mer subsets across 920 genome sequences, spanning from primates to prokaryotes. This revealed an evolutionary mechanism in genome sequences, indicating the presence of both CG and TA-specific selection modes. In the present study, we further investigate the specific selection modes in coronavirus genomic sequences by examining the intrinsic distribution rules of 32 XYi 6-mer subset spectra. Our results show that coronavirus genomes exhibit only the CG-specific selection mode, with no evidence of TA-specific selection. Using the CG-specific selection mode, we identified CG1 6-mers as the fundamental subset underlying coronavirus genome evolution. To validate the CG1 subset, we constructed phylogenetic relationships for a set of coronaviruses and SARS-CoV-2 variant genomes. Comparative analysis confirmed that the resulting phylogenetic relationships align more closely with established knowledge. This study thus provides a theoretical framework for inferring phylogenetic relationships at the whole-genome level.

## 1. Introduction

Coronaviruses are characterized as linear, positive-sense, and single-stranded RNA viruses, possessing the largest viral genomes and sequence lengths of approximately 30 kbp [1]. The family *Coronaviridae* comprises four genera: *Alphacoronavirus*, *Betacoronavirus*, *Gammacoronavirus*, and *Deltacoronavirus* [2]. It is evident that *Alphacoronavirus* and *Betacoronavirus* principally infect mammals, whereas *Gammacoronavirus* and *Deltacoronavirus* primarily infect birds [3]. Currently, seven distinct coronaviruses have been found to infect humans. Of these, HCoV-OC43, HCoV-HKU1, HCoV-NL63, and HCoV-229E are typically associated with mild symptoms reminiscent of the common cold. It has been demonstrated that children and the elderly are particularly vulnerable to developing lower respiratory tract diseases, or more severe respiratory diseases [4]. However, in 2003, the Severe Acute Respiratory Syndrome Coronavirus (SARS-CoV) caused more than 8000 infections and 774 deaths, while in 2012, the Middle East Respiratory Syndrome Coronavirus (MERS-CoV) resulted in more than 850 deaths [5]. In 2019, Severe Acute Respiratory Syndrome Coronavirus 2 (SARS-CoV-2) caused severe medical burdens and economic losses to countries around the world [6,7].

When a new virus emerges, sequence comparison is fundamental to multiple areas, such as the evolution relationship analysis [8], homologous gene searching [9], vaccine research and development [10], drug treatment [11], virus identification [12], and control of the transmission rate [13]. Within the domain of bioinformatics, two primary sequence comparison methods are recognized: alignment algorithms and alignment-free algorithms. In general, multiple sequence alignment (MSA) algorithms are commonly applied to viral genome sequence classification, and have high accuracy, such as Clustal W (version 1.8) [14], MAFFT (version 1.0) [15], and MUSCLE (version 1.0) [16]. The advent of high-throughput DNA sequencing technology has led to a proliferation of genome sequences. Multiple sequence alignment algorithms face several challenges. First, they rely on the assumption of collinearity among homologous sequences, an assumption frequently violated by viral genomes. Their dynamic evolution, characterized by frequent recombination and horizontal gene transfer (HGT), can create mosaic sequences and non-linear relationships (e.g., genome rearrangements) that standard alignment algorithms are not designed to handle. Second, the computations are typically time-consuming and memory-intensive. Additionally, the selection of alignment parameters often relies on empirical or somewhat subjective choices.

In an effort to overcome the limitations of conventional sequence alignment methods, some researchers have explored the potential of alignment-free algorithms for microbial genomes. In 2018, Jain et al. proposed FastANI, an influential alignment-free algorithm. By computing pairwise Average Nucleotide Identity (ANI) values for all prokaryotic genomes in the NCBI database, this study demonstrated clear species boundaries among prokaryotes [17]. Consequently, an increasing number of researchers are directing their attention towards the contribution of alignment-free algorithms to the phylogenetic relationships of viruses. It is evident that the non-random characteristics of k-mer frequency in genome sequence can be utilized to develop numerous alignment-free algorithms. These algorithms facilitate the conversion of sequence information into k-mer frequency, thereby enabling the analysis of the phylogenetic relationships between species. The Feature Frequency Profiles (FFP) algorithm is the most representative for constructing phylogenetic relationships at the genomic level. When the algorithm studies the phylogenetic relationships for viruses from 11 viral families comprising 142 large dsDNA eukaryote viruses, it is necessary to filter some k-mers in order to construct phylogenetic relationships consistent with the ICTV database [18]. Lee et al. hypothesized that the repetitive elements were closely related to species evolution and selected the top-50 frequency 5-mers to construct 1766 microbes’ phylogenetic relationships [19]. He et al. proposed a new alignment-free algorithm, termed Correlation Coefficient Feature Vector (CCFV), which converts each virus sequence information into a 16 × L-dimensional numerical vector, and estimates the similarity between sequences by calculating the Euclidean distance between the vectors [20]. In the calculation process, the variable parameter L is uncertain and must be determined by multiple trial-and-error tests. Sarwan Ali et al. posit that a considerable number of k-mers exhibiting equivalent frequencies in the SARS-CoV-2 variant sequences are deemed to be “redundant” motifs. These k-mers, it is asserted, contribute negligible advances in terms of the predictive capability of the classification algorithms [21]. It is evident that the aforementioned methodologies have the capacity to enhance the reliability of constructing species phylogenetic relationships subsequent to the filtration of k-mers. Nevertheless, a critical concern that persists is the inconsistency in the number of k-mers.

In recent research, machine learning algorithms have been employed to analyze the consistent k-mer feature set that constructs the evolutionary relationships of virus sequences. In their study, Solis-Reyes et al. utilized k-mer-based machine learning algorithms to investigate the phylogenetic relationships of 2784 HIV-1 sequences. They employed dimensionality reduction techniques to enhance the accuracy of classification [22]. In 2019, following the emergence of SARS-CoV-2, a significant number of researchers selected feature sets based on machine learning algorithms for the purpose of studying its classification and evolutionary relationships. For instance, Randhawa et al. determined that 7-mers constituted the optimal feature set based on the MLDSP model [23]. Similarly, Ren et al. ascertained that the 6-mers feature set exhibited the most favourable outcomes based on the kmer2vec model [24]. In addition, Dlamini et al. ascertained that the 2-mers feature set was most efficacious for classification based on the XGBOOST algorithm [25]. Although machine learning algorithms show high sensitivity and accuracy in classifying viral genomes, they fail to resolve the inconsistency in feature set selection. Many methods used to construct viral phylogenies lack a solid theoretical basis and rely on arbitrary choices of k-mers. This leads to inconsistent evolutionary trees, making them unreliable for species classification. Therefore, determining the appropriate number of k-mers for evolutionary analysis remains the foremost challenge.

A considerable body of research has been dedicated to the analysis of k-mer spectra within genome sequences, with the objective of elucidating the correlation between genome sequence composition and evolution. Initially, Xie et al. analyzed the k-mer spectra of *Haemophilus influenzae* genome sequences and their randomized sequences [26]. The results indicated that each k-mer in the randomized sequences was an equal probability, while each k-mer in the *Haemophilus influenzae* genome sequences was a motif preference. Chen et al. analyzed the 6-mer spectrum distribution characteristics of nine genome sequences and found that the 6-mer spectrum distribution is closely related to the evolution of species [27]. Subsequently, Chor et al. studied the k-mer spectra of more than 100 species from Archaea, Bacteria, and Eukaryota. The results obtained from the analysis indicated that the majority of species exhibited a unimodal k-mer spectrum, with only the tetrapods displaying a multimodal spectrum [28].

The distribution of k-mer spectra for 48 XYi (i = 0, 1, 2) subsets was analyzed across the genome sequences of 920 species, including animals, plants, fungi, eubacteria, and archaea. The results of the study indicated the presence of two distinct selection modes within the genome sequence, namely CG and TA-specific selection modes. It has been demonstrated that CGi and TAi subset k-mers play a pivotal role in the composition and evolution of the genome [29]. Based on the distribution rules of genomic k-mer spectra, we propose an evolutionary mechanism for genome sequences. Analysis revealed that, under this mechanism, 8-mers containing CG or TA dinucleotides exhibit a direct correlation with genome evolution. In contrast, those containing CG0 or TA0 show only an indirect correlation. Consequently, CG1+CG2 8-mers and TA1+TA2 8-mers were selected as feature sets to characterize the phylogenetic relationships of genome sequences. The reliability of the theoretical approach was validated in genome sequences at the genomic level in mammals, reptiles and insects [30,31]. The present study further investigates the evolution mechanism of coronavirus genomes. Our primary research objective is to identify an objective set of k-mer features that can effectively characterize viral evolution, thereby providing a theoretical foundation for constructing phylogenetic relationships at the genomic level.

## 2. Results

### 2.1. Characteristics of 6-mer Spectrum Distributions in Coronavirus Genomes

**Figure 1:**
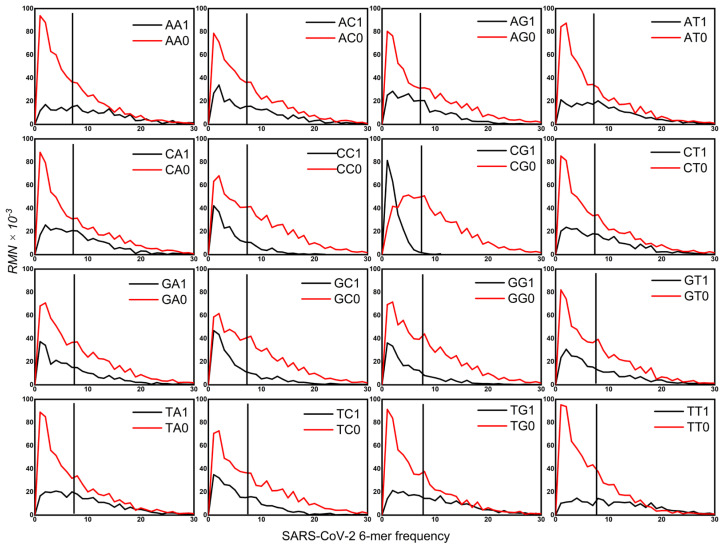
The spectra of XY1 and XY0 6-mer subsets of the SARS-CoV-2 genome sequence (X, Y = A, C, G, T). The vertical bar represents the “random center,” defined as the mean of the 6-mer frequency distribution calculated from randomized sequences that preserve the base composition of the original genome. This center corresponds to the null hypothesis of no selection. For 6-mers with zero frequency, we set *RMN* = 0. The raw data for Figure 1 are provided in Supplementary Table S3. The y-axis represents the 6-mer frequency calculated across the genome. The analysis was performed on the SARS-CoV-2 reference genome (Wuhan-Hu-1, NC_045512).

In the preceding study, an investigation was conducted into the distribution of k-mer spectra across a total of 920 genome sequences, ranging from primates to prokaryotes. This analysis enabled the identification of the evolutionary mechanism of genome sequences. The evolutionary mechanism of genome sequences reveals two specific selection modes, termed CG-specific selection and TA-specific selection. It is evident that primates, rodents, other mammals, other vertebrates, and yeasts have a strong CG-specific selection mode. In contrast, plants, non-vertebrates, and some prokaryotes have a strong TA-specific selection mode. This study builds on the findings of previous research, extending the investigation to the specific selection modes operating on coronavirus genome sequences. A thorough investigation into the spectrum distributions of 32 XYi (i = 0, 1) 6-mer subsets in coronavirus genomes (see Figure 1) has revealed that these distributions comply with the three defining characteristics of the CG-specific selection mode. One is the evolutionary separability. It is only under the CG classification that the spectrum distributions of CG1 and CG0 6-mer subsets are separated from each other. Another is the evolutionary selectivity. In comparison to the random center, the most probable frequency of CG0 6-mers was found to be in proximity to the random center, whereas that of CG1 6-mers was lower than the random center. The results of this study suggest that CG0 6-mers may arise primarily through random selection, while CG1 6-mers are likely shaped by directional selection. The third is the evolutionary conservatism. The spectrum distribution of CG1 6-mers is more conservative than that of CG0 6-mers. As illustrated in Figure 1, the spectrum distribution of 32 XYi 6-mers subsets of the SARS-CoV-2 genome sequence is depicted according to the XY dinucleotide classification method (see Section 4). The spectrum distributions of 32 XYi 6-mer subsets of the other coronaviruses’ genomes exhibited a high degree of similarity to that of SARS-CoV-2.

### 2.2. Distribution Map of Specific Selection Intensity

**Figure 2:**
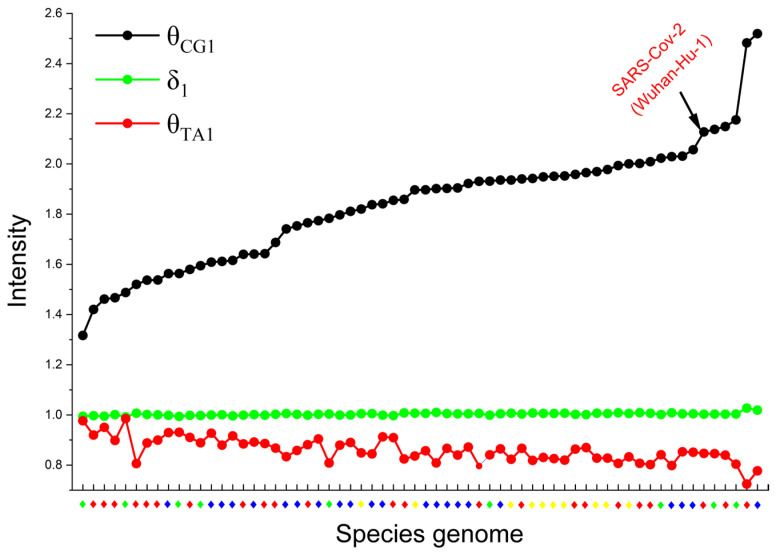
Distribution map of specific selection intensity. The relationship between intensity distributions of CG and TA-specific selection in 64 coronavirus genome sequences. *Alphacoronavirus* (blue rhombus), *BetaCoronavirus* (red rhombus), *GammaCoronavirus* (yellow rhombus), and *DeltaCoronavirus* (green rhombus). The metrics $\theta_{CG1}$ and $\theta_{TA1}$ represent the intensities of CG-specific selection and TA-specific selection, respectively. The reference value $\delta_{1}$ denotes the average separability of the remaining 14 XY1 6-mer subsets (excluding CG1 and TA1).

To quantitatively characterize the specific selection modes of CG and TA in the 6-mer spectrum distribution of coronavirus genomic sequences, we propose a separability feature parameter. For a given XYi 6-mer subset spectrum, $\delta_{XYi}$ represents its separability value. Notably, our earlier research revealed that the evolutionary information of genome sequences is mainly reflected in CG and TA motifs [29]. Consequently, we decided to use $\theta_{CG1}$ to represent CG-specific selection intensity and $\theta_{TA1}$ to represent TA-specific selection intensity. For ease of comparison, the average value of the separability ($\delta_{1}$) of the remaining 14 kinds of XY1 6-mer subsets (excluding CG1 and TA1) is used as a reference value. In coronavirus genomes, when $\theta_{CG1}>\delta_{1}$, this indicates a strong CG-specific selection mode. Similarly, a finding that $\theta_{TA1}>\delta_{1}$ indicates a strong TA-specific selection mode. Figure 2 shows the intensity distribution of CG and TA-specific selection for 64 coronavirus genome sequences. Genomes are arranged along the x-axis in ascending order of $\theta_{CG1}$ values, with corresponding genome names and sorting order provided in Supplementary Table S3. This finding indicates that the CG-specific selection mode was strong, whereas the TA-specific selection mode was weak in coronavirus genomes.

Previous studies have shown that the intensity of CG-specific selection can reflect the rate of evolution of a species’ genome [29]. Accordingly, a $\theta_{CG1}$ value lower than the species average indicates slow evolution, whereas a higher value indicates rapid evolution. As illustrated in Figure 2, the three leftmost genome sequences of the coronavirus are Magpie robin coronavirus HKU18 (avian coronavirus), Rousettus bat CoV (bat coronavirus), and Pipistrellus bat CoV HKU5 (bat coronavirus). These three sequences demonstrate the lowest CG-specific selection intensity. This finding suggests that these three coronaviruses are relatively ancient and exhibit a reduced rate of evolution. This result is consistent with the findings of virologists. For example, Lau et al. found that three novel *Deltacoronaviruses* originated from Magpie robin coronavirus HKU18 [32], and Lau et al. found that Pipistrellus bat CoV HKU5 is the ancestor of MERS-CoV [33]. As shown in Figure 2, two human coronaviruses (Human CoV HKU1 and NL63) exhibited the highest CG-specific selection intensity. This suggests that they are evolving rapidly within the coronavirus group.

In summary, Figure 1 and Figure 2 demonstrate that the coronavirus genomes examined here exclusively exhibit the CG-specific selection mode. Building on this observation, we hypothesize that the 6-mers containing CG dinucleotides may act as a key driver of coronavirus evolution. To test this hypothesis, we will analyze representative sequences from diverse coronaviruses and SARS-CoV-2 variants in the following sections.

### 2.3. Phylogenetic Relationship of Coronavirus

The findings from the previous section indicate that coronavirus genomes exhibit only the CG-specific selection mode, while the TA-specific selection mode is absent (Figure 2). This suggests that the CG1 6-mers constitute a core subset for coronavirus genome evolution. To assess the validity of the CG1 6-mers subset, the total 6-mers were divided into three subsets: (1) CG1 6-mers (N_1_ = 1185), (2) CG0 6-mers (N_0_ = 2911), and (3) CG1+CG0 6-mers (N_1_ + N_0_ = 4096). Following the aforementioned method, distance matrices were constructed, and phylogenetic relationships among different coronavirus genomes were inferred using MEGA 12 software. To validate the accuracy of the inferred relationships, a reference phylogeny was constructed using the maximum-likelihood method based on a multiple sequence alignment generated with MAFFT (v7.526).

**Figure 3:**
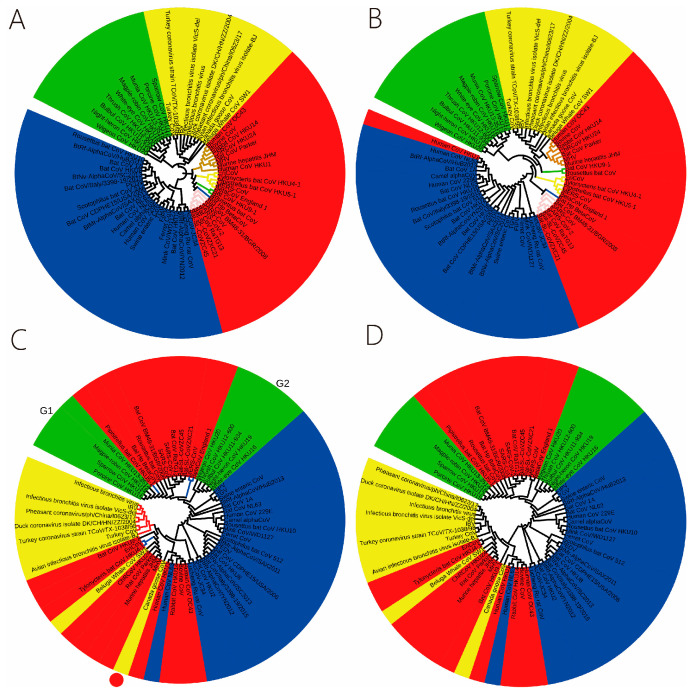
Phylogenetic relationships of coronavirus genome sequences. (**A**) Reference phylogenetic relationship (based on the maximum-likelihood method), (**B**) The phylogenetic relationship is constructed by CG1 6-mers. (**C**) The phylogenetic relationship is constructed by CG0 6-mers, (**D**) The phylogenetic relationship is constructed by CG1+CG0 6-mers. The 4 color backgrounds represent the different genera of Coronoviridae: *Alphacoronavirus* (blue), *Betacoronavirus* (red), *Gammacoronavirus* (yellow), and *Deltacoronavirus* (green).

The phylogenetic relationships of coronavirus genomes were obtained from the three subsets, with the results displayed in Figure 3B–D. The reference phylogenetic relationship is shown in Figure 3A. The viral genomes are classified into four genera, as illustrated in Figure 3: *Alphacoronavirus* (blue background), *Betacoronavirus* (red background), *Gammacoronavirus* (yellow background), and *Deltacoronavirus* (green background). A comparison of Figure 3A,B shows that the phylogeny constructed using CG1 6-mers is consistent with that obtained by the maximum-likelihood method. The phylogenetic relationship inferred from CG1 6-mers aligns with the established taxonomy of coronaviruses. However, a notable exception is the classification of human CoV-HKU1. Although it belongs to the *Betacoronavirus* genus, it clusters phylogenetically with human CoV-NL63, which is an *Alphacoronavirus*. This anomalous placement may be attributed to the similarity in the overall periodic structure between human CoV-HKU1 and human CoV-NL63 [24]. This problem merits further study. In essence, the phylogenetic relationship derived from the CG1 6-mers is biologically meaningful. This is exemplified within the *Betacoronavirus* genus, where the CG1 6-mers analysis supports the division into five established subgenera: *Sarbecovirus* (pink clade), *Hibecovirus* (blue clade), *Nobecovirus* (green clade), *Embecovirus* (brown clade), and *Merbecovirus* (yellow clade).

A comparison of Figure 3A,C reveals that the phylogenetic relationship inferred from the CG0 6-mers is incongruent with the reference phylogeny. The main discrepancies are as follows: (a) Within *Betacoronavirus*, the resolution at the subgenus level is poor. For instance, the *Merbecovirus* subgenus is divided into three evolutionary clades (blue clades, Figure 3C) and the three clades are far apart. (b) Within *Gammacoronavirus*, Canada goose coronavirus (red circle, Figure 3C) is erroneously placed as an outgroup to the main avian coronavirus cluster (red clades, Figure 3C). This finding stands in contrast to the previously established conclusion that avian coronaviruses share a common progenitor. (c) The nine *Deltacoronavirus* sequences are split into two distantly related clades (G1 and G2, Figure 3C), a topology that is inconsistent with the established monophyly of this genus.

The findings indicate that the CG1 6-mers are directly associated with the evolution of the coronavirus genome, while the CG0 6-mers exhibit an indirect relationship with genome evolution. Consequently, the CG0 subset does not provide new evolutionary information. The phylogenetic relationship constructed using CG1+CG0 (total 6-mers) is demonstrated in Figure 3D. A comparison between Figure 3A,D reveals that the phylogeny inferred from the combined set shows poor congruence with the reference phylogeny. This discrepancy is attributed to the informational redundancy and substantial background noise introduced by the CG0 6-mers, which compromises phylogenetic accuracy (Figure 3D). Therefore, we conclude that the CG1 6-mers subset is the most informative and optimal feature set for constructing accurate phylogenetic relationships of coronavirus genomes.

### 2.4. Phylogenetic Relationship of SARS-CoV-2 Variants

With the emergence of SARS-CoV-2 variants, researchers have focused on them for two main reasons: to accurately and timely monitor the virus’s evolution, and to support vaccine research and development.

**Figure 4:**
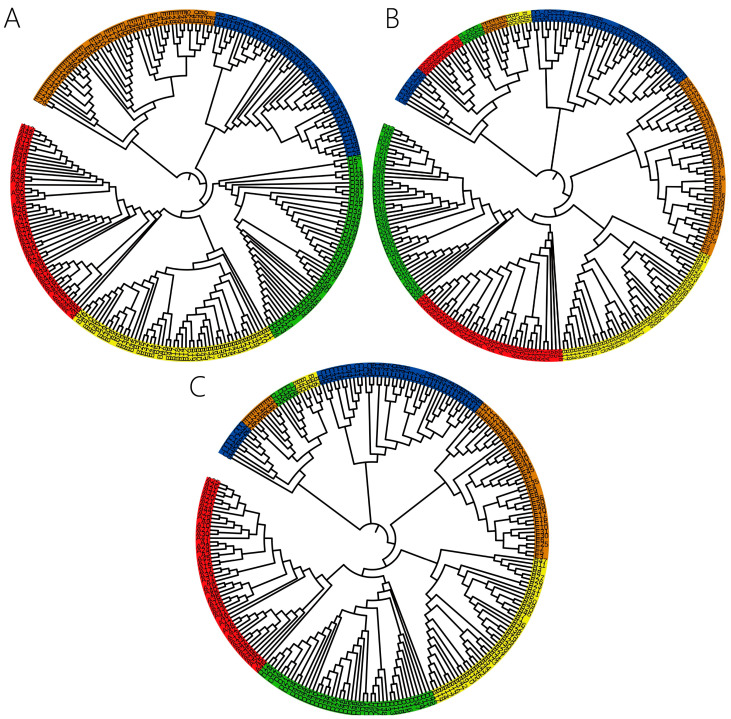
Phylogenetic relationships of the five SARS-CoV-2 variants. (**A**) The phylogenetic relationship is constructed by CG1 6-mers, (**B**) The phylogenetic relationship is constructed by CG0 6-mers, (**C**) The phylogenetic relationship is constructed by CG1+CG0 6-mers. The red background represents the alpha variant, the yellow background represents the beta variant, the green background represents the gamma variant, the blue background represents the delta variant, and the brown background represents the omicron variant.

We selected five major SARS-CoV-2 variants from GISAID: Alpha, Beta, Gamma, Delta, and Omicron. To ensure data reliability and analytical accuracy, we applied the following quality control criteria: (i) only complete genome sequences with high coverage were included; (ii) sequences were excluded if they contained more than 5% ambiguous bases (‘N’s) or were associated with metadata flags such as ‘low-coverage’ or ‘partial’. Correspondingly, the total 6-mers were partitioned into three feature subsets: (1) CG1 6-mers (N_1_ = 1185), (2) CG0 6-mers (N_0_ = 2911), and (3) CG1+CG0 6-mers (N_1_ + N_0_ = 4096). To validate the effectiveness of these feature subsets, we constructed the phylogenetic relationships among these variants using each subset. The resulting phylogenies are presented in Figure 4.

In the phylogenetic relationships, different SARS-CoV-2 variants are color-coded. Effective clustering is indicated when sequences of the same color form monophyletic groups. Overall, the phylogeny inferred from the CG1 6-mers performs well in this regard (Figure 4A). In contrast, the CG0 6-mers fail to resolve the five major variants into distinct monophyletic clades. For instance, in Figure 4B, sequences from multiple variants (e.g., 5 Beta, 6 Gamma, 5 Delta, and 7 Omicron) are incorrectly grouped into a single, spurious clade. This erroneous grouping contradicts the well-established phylogenetic distinction among these variants. The phylogeny generated from the combined CG1+CG0 set also shows poor resolution (Figure 4C). To quantitatively assess clustering accuracy, we employed the monophyletic consistency score ($Q_{m}$), which indicated that most established taxonomic groups formed distinct, pure clades in the 6-mer-based phylogeny. The $Q_{m}$ value was 0.8 for the phylogenetic relationships inferred from the CG1 subset, 0 for the CG0 subset, and 0.2 for the combined CG1+CG0 set. Based on this analysis, we confirm that the CG1 6-mers constitute the optimal feature set for constructing accurate phylogenies of SARS-CoV-2 variant genomes.

## 3. Discussion

The findings indicate that the sequence analysis method employed in this study—based on a subset of 6-mer CG1 features—is effective for coronavirus genome comparison and supports the construction of phylogenetic relationships. This method successfully classified the studied coronaviruses into four major clades and further subdivided the genus *Betacoronavirus* into five subgenera: *Sarbecovirus*, *Hibecovirus*, *Nobecovirus*, *Embecovirus*, and *Merbecovirus*. This classification result has been demonstrated to align with existing research findings [34,35], thereby validating the reliability of the method. A plethora of studies have indicated that Bat_CoV_RaTG13 shares a close phylogenetic relationship with SARS-CoV-2, and both exhibit similar characteristics in genomic G+C content and CpG dinucleotide frequency [36]. Our phylogenetic analysis derived from the CG1 6-mer subset places Bat_CoV_RaTG13 and SARS-CoV-2 on sister clades, firmly establishing their closest evolutionary affinity. By analyzing the distribution rules of 6-mer spectra in coronavirus genomes, this study identified a feature subset (CG1) that is closely associated with viral evolution, thereby effectively enhancing the accuracy of phylogenetic construction. Despite minor discrepancies between the phylogenetic placement of Human_CoV_HKU1 constructed by our method and the reference phylogeny, these may be attributable to the continuous evolution of coronaviruses. For instance, Konishi et al. [37] identified significant evolutionary divergence of Human_CoV_HKU1 within the *Embecovirus* subgenus via principal component analysis (PCA). In accordance with this observation, our study also noted a significantly higher CG-specific selection intensity in Human_CoV_HKU1 in comparison to other *Betacoronavirus* members (Figure 2), indicating a potentially accelerated evolutionary rate for this virus. This result corroborates the findings from PCA, thereby enhancing the credibility of our conclusions and providing a reasonable explanation for the subtle discrepancies observed in phylogenetic analyses.

A significant challenge in coronavirus genomics is identifying k-mer feature sets that correlate with evolutionary patterns. Notably, k-mers containing the CG dinucleotide in RNA virus genomes are frequently associated with viral evolution. For instance, when influenza viruses transitioned from avian to human hosts, their genomes exhibited a gradual reduction in CG motifs under selective pressure [38]. Digard et al. found that CpG suppression in coronavirus genomes varies with host species and tissue tropism [39]. Furthermore, experimentally increasing CG dinucleotides has been shown to consistently reduce viral replication and virulence, as demonstrated in studies on HIV-1 [40], Echovirus [41], and Zika virus [42]. Kumar et al. reported that SARS-CoV-2 variants of concern (Alpha, Beta, and Delta) harbored fewer CG motifs compared to early pandemic sequences [43]. Collectively, these lines of evidence point to CG motifs as critical determinants of viral evolution and host adaptation. Building on this foundation, we identify an objective 6-mer feature set—termed the CG1 subset—that effectively characterizes coronavirus genome evolution. By integrating these prior findings with our results, we propose that CG motifs function as key evolutionary drivers in coronaviruses, thereby offering a new framework for deciphering their evolutionary patterns.

It is widely acknowledged that birds and bats act as the natural reservoirs of coronaviruses [44]. The precise origins of coronaviruses remain uncertain; it is unclear whether they first emerged in bats and subsequently transmitted to birds or vice versa. Bats are considered optimal hosts for *Alphacoronaviruses* and *Betacoronaviruses*, while birds are regarded as suitable hosts for *Gammacoronaviruses* and *Deltacoronaviruses*. In the bat coronavirus lineage, bat coronaviruses have been observed to jump to other bat species and other mammals, including humans. Each jump between species appears to promote virus evolution. In a similar manner, within the avian coronavirus lineage, the avian coronavirus is transmitted to another bird, thereby giving rise to the new *Gammacoronavirus* and *Deltacoronavirus* [45]. It is evident that coronaviruses undergo evolution in the course of each interspecies transmission. The CG-specific selection intensity distribution map for 64 coronavirus genomes (see Figure 2) provides a substantial amount of image information. It is posited that these images not only present the current status of the evolution of coronavirus genome sequences, but also the evolutionary status and process of coronavirus genome sequences in early life. In this study, we draw a parallel with astronomical methods to hypothesize about the evolution of coronavirus genomes. Just as astronomers infer stellar evolution by observing stars at different life stages, we can infer viral evolutionary processes by analyzing genomic sequences representing different evolutionary states. In our CG-specific selection intensity distribution map, when the abscissa represents different coronavirus genomes, the map captures their current evolutionary states. Conversely, when the abscissa is interpreted as a continuous axis of genomic change, the map can be viewed as a representation of the evolutionary trajectory of coronaviruses from the past to the present.

A widely used approach for comparing viral genome sequences is to calculate genetic distances based on the multi-sequence alignment of a single conserved gene, such as the Coronavirus RdRp gene [46]. However, evolutionary scientists argue that a single gene can only represent partial evolutionary information. For example, the RdRp gene is approximately 387 base pairs long, accounting for merely 1.29% of the ~30 kilobase SARS-CoV-2 genome. Consequently, evolutionary analyses based solely on such a gene fail to capture genome-wide information, which may limit the accuracy of inferred phylogenetic relationships. The advent of high-throughput sequencing has led to a rapid accumulation of whole genome sequences in public databases. While whole-genome alignment could theoretically resolve the issue of incomplete coverage, it is computationally challenging due to the NP-complete nature of the problem. Alternatively, alignment-free methods based on k-mers have been extensively adopted for comparative viral genomics. However, phylogenetic relationships derived from the complete set of k-mers are often unsatisfactory. Many researchers have attempted to improve results by filtering high- or low-frequency k-mer sets, which indeed enhances the quality of evolutionary reconstructions. Yet, a critical challenge remains: the inconsistent number of k-mers retained across studies. To address this, our method systematically distinguishes evolutionarily sensitive from insensitive k-mer sets and provides a theoretical basis for selecting comprehensive yet informative k-mer features, thereby improving the robustness of evolutionary analyses.

**Figure 5:**
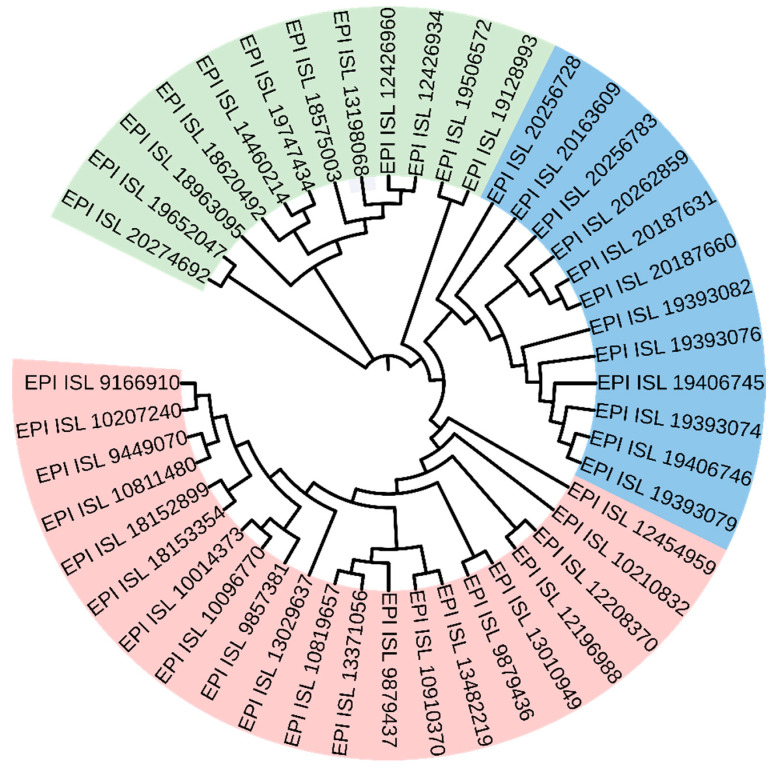
Phylogenetic relationship of XD recombinants, delta, and omicron. The light pink background represents the XD recombinants, the light blue background represents the delta variant, the light green background represents the omicron variant.

The concurrent transmission and co-infection of multiple coronavirus variants can trigger genetic recombination, a mechanism central to viral evolution research [47,48,49,50]. To accurately resolve phylogenetic conflicts caused by recombination, this study proposes a phylogenetic method based on a subset of CG1 features extracted from the whole-genome 6-mer spectrum. This approach filters noise from the total 6-mer set, retaining only the 6-mers that are directly related to evolution. We focused on the XD recombinant (also known as “Deltacron”; Pango lineage XD), whose genome is largely derived from the Delta variant but incorporates the spike gene from Omicron [51]. As shown in Figure 5, the phylogenetic tree constructed with the CG1 subset clearly separates SARS-CoV-2 variants into three distinct clades: XD recombinants, delta, and omicron. Topologically, XD recombinants cluster monophyletically with delta, a finding that is consistent with their evolutionary background in which delta serves as the major parental lineage. These results confirm the efficacy of our method in identifying and resolving phylogenetic conflicts due to recombination, thereby offering a novel methodological framework for analyzing viral genetic recombination.

Genome evolution is governed by fundamental molecular mechanisms that may operate across diverse life forms. Building on our prior elucidation of these mechanisms in eukaryotic genomes, we extended the investigation to viral systems, specifically focusing on coronaviruses and SARS-CoV-2 variants to explore their genome evolution. A thorough investigation into the distribution rules of 32 XYi 6-mer subsets of spectra in coronavirus genome sequences was conducted, resulting in the conclusion that their genomes exhibited exclusively the CG-specific selection mode, while the TA-specific selection mode had become extinct. The utilization of the CG-specific selection mode resulted in the identification of a 6-mer subset that exhibited a substantial association with the genomic evolution of coronaviruses. The analysis indicated a direct correlation between 1185 6-mers in the CG1 subset and the evolution of the coronavirus genome. To further validate these findings, we constructed phylogenetic relationships for both coronaviruses and SARS-CoV-2 variants. Following rigorous analysis and comparison, it was determined that the phylogenetic relationships are consistent with the established conclusions.

## 4. Materials and Methods

### 4.1. Coronavirus Genome Sequences

Coronavirus genome sequences were obtained from NCBI (https://www.ncbi.nlm.nih.gov/, accessed on 6 April 2026) and GISAID (https://www.gisaid.org/, accessed on 6 April 2026). The detailed information is shown in Supplementary Table S1.

### 4.2. Variants of SARS-CoV-2 Genome Sequences

Complete, high-coverage 250 sequences for SARS-CoV-2 variants were randomly extracted from GISAID. We selected five major variants (alpha, beta, gamma, delta, and omicron) from the 10 countries and different laboratories. Each variant contains 50 genome sequences. The SARS-CoV-2 variant sequences detailed information was shown in Supplementary Table S2.

### 4.3. Selection of k Value

In the k-mer spectra analysis, selecting appropriate k values will significantly affect the effectiveness of the feature set. It is important to note that short k values may not capture enough evolutionary information, while long k values are susceptible to sequence length and noise interference. In coronavirus genomes, the k value is selected as 6. The reasons are as follows: Beny Chor [28] proposed a formula $k=\left\lceil {0.7\log}_{4}L\right\rceil$ to estimate the most appropriate *k* value, *L* is the length of the given genome sequence. The length of the coronavirus genome sequence is about 30 kbp and the calculated *k* value is 6. Accordingly, we chose *k* = 6 for our study.

### 4.4. 6-mer Spectral Distribution

No preprocessing steps (e.g., removal of ambiguous bases, length normalization, or filtering of incomplete genomes) were applied to the genome sequences prior to analysis. For each coronavirus genome sequence of length L base pairs (bp), the occurrence frequencies of all possible 6-mers (4096 in total) are calculated using a sliding window approach with a window size of 6 bp and a step size of 1 bp. If the number is $N_{i}$ for the 6-mers with frequency *i*, the relative motif number (*RMN*) in the frequency block *i* is defined as the following:

(1) $$RMN=\frac{N_{i}}{4^{6}}$$

With the 6-mer frequency as the abscissa and the relative motif number (*RMN*) as the ordinate, the distribution of the relative motif number (*RMN*) with the 6-mer frequency is obtained, which is called the 6-mer spectrum.

### 4.5. XY Dinucleotide Classification Method

For each coronavirus genome sequence, we obtained its total 6-mers (4^6^ = 4096). Based on the presence or absence of an XY dinucleotide (where X, Y ∈ {A, T, C, G}) in the motif, the XY dinucleotide classification method categorizes the total 6-mers into two distinct groups: those containing the XY dinucleotide (designated as XY1) and those lacking it (designated as XY0).

When X ≠ Y, there are 2911 XY0 6-mers and 1185 XY1 6-mers. When X = Y, there are 3105 XY0 6-mers and 991 XY1 6-mers. Thus, in 16 kinds of XY dinucleotide classification, 32 XYi (i = 0, 1) 6-mer subsets are obtained from the total 6-mers.

### 4.6. Separability of a Spectrum

To quantify the positional distribution differences between the spectrum of a given 6-mer subset and the spectrum of total 6-mers in a genome sequence, we define the separability as follows:

(2) $$\delta_{i}=\frac{\bar{x}}{{\bar{x}}_{i}}$$

Here, $\bar{x}$ represents the average frequency of the total 6-mers, called the random center. ${\bar{x}}_{i}$ represents the average frequency of the 6-mers in the *i*th 6-mer subset. The $\delta_{i}$ represents the degree of separation from the spectrum position of the *i*th subset relative to the random center. If $\delta_{i}$ > 1, that means the spectrum of the *i*th k-mer subset is located at the lower frequency end and is away from the random center. The larger $\delta_{i}$ is, the farther the 6-mer subset spectrum is from the random center, and the greater the separability. The feature parameter $\delta_{i}$ can be used to compare the relative separability of 6-mer spectra not only among different subsets within a single genome sequence but also among different genome sequences. Biologically, $\delta_{i}$ represents the divergence in evolutionary status between the compared sequences, or more specifically, the degree of structural divergence.

### 4.7. Construction of Phylogenetic Relationship

For a set of coronavirus genome sequences $S=\{S_{1},S_{2},\ldots ,S_{k}\}$, this study employs an evolutionary distance metric based on 6-mer frequency to quantitatively assess genetic divergence between sequences. Specifically, the evolutionary distance between two sequences $S_{i}$ and $S_{j}$ is defined as:

(3) $$D_{ij}=\sqrt{\frac{\sum_{k=1}^{N}{\left(x_{ik}-x_{jk}\right)}^{2}}{N}}$$

(4) $$x_{k}=\frac{m_{k}}{\sum_{l=1}^{N}m_{l}}$$

Here, $x_{ik}$ and $x_{jk}$ denote the relative frequencies of the *k*-th 6-mer in sequences $S_{i}$ and $S_{j}$, respectively; $m_{k}$ is the frequency of the *k*-th 6-mer, and *N* is the total number of selected 6-mer features. When the total 6-mer set is selected, *N* = 4096; when the CG1 6-mer set is selected, *N* = 1185; when the CG0 6-mer set is selected, *N* = 2911. This distance metric effectively captures global differences in 6-mer composition patterns between sequences, providing a quantitative measure of their evolutionary divergence.

(5) $$D_{s}=\left[\begin{array}{ccc} D_{11} & \ldots & D_{1k}\\ \vdots & \ddots & \vdots \\ D_{k1} & \ldots & D_{kk} \end{array}\right]$$

Based on the aforementioned distance definition, this study constructed a symmetric distance matrix $D_{s}$ for the sequence set S, where each element $D_{ij}=d(S_{i},S_{j})$ denotes the evolutionary distance between sequences $S_{i}$ and $S_{j}$. Phylogenetic relationships among the sequences were inferred using the Neighbor-Joining (NJ) method applied to $D_{s}$. The resulting NJ tree illustrates the genetic relatedness of the viral genomes and was visualized with MEGA software (version 12.0).

### 4.8. Monophyletic Consistency Score

For each annotated group $g_{i}$ with sequence set $S_{i}$, we defined the clade derived from the lowest common ancestor ${LCA(S}_{i})$ as ${C(LCA(S}_{i}))$. A group was strictly monophyletic if ${C(LCA(S}_{i}))⊑S_{i}$, coded by an indicator function $I(g_{i})=1$; otherwise $I(g_{i})=0$. The monophyletic consistency score was calculated as:

(6) $$Q_{m}=\frac{1}{N}\sum_{i=1}^{N}I(g_{i})$$

where N is the total number of studied groups.

## 5. Conclusions

- Coronavirus genomes exhibit only the CG-specific selection mode, with no evidence of TA-specific selection.
- We identified an optimal set of 6-mer features that effectively characterizes coronavirus genome evolution, providing a theoretical foundation for constructing phylogenies at the genomic level.

## Data Availability

Coronavirus genome sequences and SARS-CoV-2 variant sequences were obtained from NCBI (https://www.ncbi.nlm.nih.gov/, accessed on 6 April 2026) and GISAID (https://www.gisaid.org/, accessed on 6 April 2026). The original contributions presented in this study are included in the article/Supplementary Materials. Further inquiries can be directed to the corresponding author.

## Supplementary Materials

The following supporting information can be downloaded at: https://www.mdpi.com/article/10.3390/ijms27083604/s1.
